# Longitudinal analysis of long-term outcomes of colorectal cancer after laparotomy and laparoscopic surgery: The Shizuoka study

**DOI:** 10.1371/journal.pone.0294589

**Published:** 2023-11-17

**Authors:** Noriko Kojimahara, Yasuto Sato, Yoko Sato, Fumihiro Kojimahara, Katsuyuki Takahashi, Eiji Nakatani

**Affiliations:** 1 Research Support Center, Shizuoka General Hospital, Shizuoka, Japan; 2 Graduate School of Public Health, Shizuoka Graduate University of Public Health, Shizuoka, Japan; 3 School of Medicine, Keio University, Tokyo, Japan; 4 Takahashi Gastroenterology Clinic, Saitama, Japan; Tehran University of Medical Sciences, ISLAMIC REPUBLIC OF IRAN

## Abstract

**Background:**

Long-term cancer prognosis after initial surgical procedures is an unlikely endpoint for clinical trials. Medical claim databases may aid in addressing this issue regardless of limited information on disease and patient background. However, the long-term prognosis (especially regarding long-term care needs) following surgical procedures remains unclear. This study aimed to assess whether long-term outcomes, such as the exacerbation of long-term care needs and mortality, differ with surgical methods.

**Methods:**

Using a longitudinal study with linkage between medical claim and long-term care database, patients with primary colorectal cancer who underwent initial colonoscopies were identified through anonymized data in Japan (Shizuoka Kokuho Database, 2012–2018). Odds ratios (ORs) for long-term outcomes (long-term care needs and all-cause mortality during a 6.5-year follow-up period) were analyzed using logistic regression to compare laparoscopy and endoscopic surgery to laparotomy.

**Results:**

Overall, 3,744 primary colorectal cancer cases (822 laparotomies, 705 laparoscopies, and 2,217 endoscopic surgeries) were included. Compared to the laparotomy group, the crude OR for exacerbation of long-term care needs in the laparoscopic surgery group was 0.376 (95% confidence interval, 0.227, 0.624), while the OR for all-cause mortality was 0.22 (0.329, 0.532).

**Conclusion:**

This is the first study to analyze long-term prognosis after surgery for patients with colorectal cancer to combine medical and long-term needs data. As the national health insurance claim database rarely includes information on cancer stage and comorbidities, better prognosis on endoscopic surgery may need careful interpretation. Therefore, laparoscopy has superior outcomes in terms of long-term care needs and mortality compared to those of laparotomy.

## Introduction

The incidence of colorectal cancer (CRC) ranks second after that of lung cancer in Japan. Although the age-adjusted incidence rate is currently trending towards a decrease, 158,500 patients and 54,000 deaths were reported in recent annual reports [1, 2]. Concurrently, the number of people requiring support or long-term care has continued to rise, reaching 6.674 million (2.105 million men and 4.569 million women) [3]. Approximately 18.4% of those aged ≥65 years require primary insured long-term care [4], and the number of older people with both medical and long-term care needs has increased [5]. Utsumi et al. previously reported that medical costs were substantially lower in patient groups with curable CRC than in non-curable groups using nationwide medical claims data in Japan [6]. Kurita et al. [7] examined the association of the results of certain blood tests and medical examinations with long-term care needs. However, to the best of our knowledge, no study on cancer has used data from the Shizuoka Kokuho Database (SKDB).

According to a descriptive report from the Japan Cancer Registry in 2018, of the 24,611 registered patients with Stage I CRC, the distribution of first treatment regimen ratios was as follows: 26% received endoscopic surgery, 9% received laparotomy, and 58% received laparoscopy; among patients with Stage 0 CRC, >90% received endoscopic surgery [8]. Although clinical trials have suggested the benefits of laparoscopic surgery compared with open surgery [9–11], the long-term prognosis (especially regarding long-term care needs) following surgical procedures remains unclear. To date, even medical studies based on big data rarely identified the factors exacerbating long-term care needs in patients hospitalized for cancer treatment [12, 13].

This study aimed to investigate whether laparoscopic surgery was associated with a better long-term prognosis regarding the exacerbation of long-term care needs and mortality compared with laparotomy in patients with primary CRC using SKDB, linkage medical claim, and long-term care database.

## Material and methods

### Population and study setting

This was a retrospective cohort study using the SKDB administered by prefectural National Health Insurance (NHI) organizations [13]. Shizuoka is one of the first prefectures in Japan to obtain municipal approval for person-level linkage among anonymous medical claims, health check-ups, and long-term care data [14–18]. Data including sex, age, dates of access to the NHI and latter-stage elderly medical insurance system (LEMIS) [19], as well as a record of the first health checkup before surgery for the initial evaluation of CRC, were extracted between the fiscal year of 2012–2018. Data that did not include sex and age were excluded from the original SKDB database, and the details thereof, including information on the data cleaning methods used in the study and linkage quality evaluation, have been published elsewhere [20].

### Case definition and data extraction

**Fig 1:**
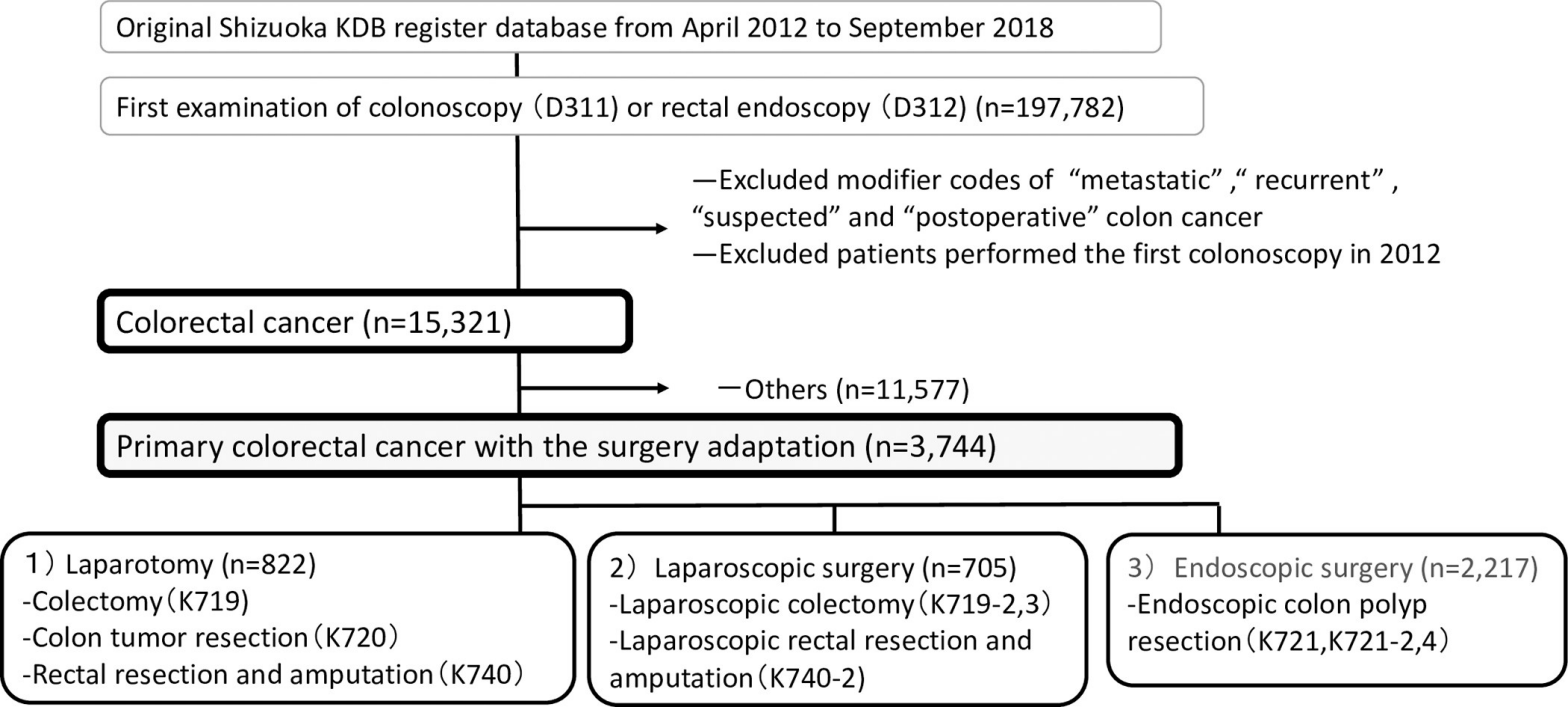
Flowchart for participant inclusion. Note that the Japanese fiscal year was defined as extending from April to March.

**Fig 1** shows the data for 197,782 patients who underwent colonoscopy (D311) or rectal endoscopy (D312). Cases with modifier codes metastatic, recurrent, suspected, and postoperative CRC were excluded from the anonymized KDB data (3088–3091, 3118, 4025, 4026, 7537, 8002, 8015–8017, 8048, and 8057). Data on patients who underwent their first colonoscopy in 2012 were deleted to designate a disease-free period before the initial diagnosis. The final study was set for a 6.5-year follow-up period from January 2013 to September 2018.

Based on the medical summary procedural codes shown in **S1 Table,** the initial procedures for primary CRC following initial colonoscopy or rectal endoscopy were classified into three groups: laparotomy, laparoscopy, and endoscopic surgery. An overlap of the surgical procedures was not considered in this study. Patients who underwent colorectal surgery with more invasive procedures, such as combined resection of other organs and those who received therapy other than surgical therapy were classified into the “others” group and were excluded from the final analysis.

### Outcomes

The primary evaluated outcome was an exacerbation of long-term care needs during a 6.5-year follow-up period following initial cancer surgery. In the Japanese LEMIS, an older adult in need can receive the necessary care service by self-pay (10–30%) at age ≥65 years [19]. In case they apply, care needs are judged every 2 years: not applicable, requiring support 1, requiring support, and 2, requiring long-term care (levels 1 [the stage when the need for care is lowest] to 5 [bedridden]). In this study, long-term care exacerbation was defined as at least one degree worse in requiring support (1 and 2) or long-term care (levels 1 to 4) following initial CRC surgery than before the surgery. As true long-term care level at the surgery was not included in the KDB dataset, 5 cases of Level 5 before the patient was diagnosed with the primary CRC were also considered as long-term care exacerbation.

The secondary outcome evaluated herein was all-cause mortality. Information on this outcome was extracted from the “reasons for death withdrawal” provided in the NHI and LEMIS records. Both outcomes were followed up for 6.5 years in this claims database.

### Statistical analysis

Given traditional assumptions (80% power; α = 0.05), a *post hoc* power analysis indicated that the final sample size (n = 3,744) was large enough to detect at least a medium-size effect of r = 0.3. Wilcoxon rank sum tests were used to compare continuous variables, and Chi-square tests were used to compare categorical variables. Odds ratios (ORs) and 95% confidence intervals (CIs) were calculated using logistic regression analysis with the laparotomy group as the reference. Age and sex were adjusted for Model 2, while possible confounders such as smoking status, alcohol consumption, physical activity, and significant variables at the baseline such as BMI, serum hemoglobin (Hb), and cardiovascular disease were adjusted for Model 3. Variables with a relatively large number of missing values in the health checkup data were excluded. As missing covariates did not occur completely at random among all participants, a simple missing data imputation was not carried out. Data from the SKDB were imported using SAS statistical software (v.9.4, SAS Institute Inc., Cary, NC, USA), and all analyses were performed using STATA statistical software (v.16, College Station, TX, USA).

All SKDB data were anonymized to protect participant confidentiality. Thus, informed consent from each enrollee was not required for this study [20]. This study protocol was approved by the ethics committee of Shizuoka Graduate University of Public Health (#SGUPH_2021_001_0009). This study was conducted in accordance with the principles of the Declaration of Helsinki. This manuscript was evaluated by the Authors using the RECORD checklist [21] (**S2 Table**).

## Results

### Baseline characteristics

In total, 3,744 patients with primary CRC receiving surgery for inclusion in this study were identified: 822 patients who underwent laparotomy, 705 patients who underwent laparoscopic surgery, and 2,217 patients who underwent endoscopic surgery (**Fig 1**). **Table 1** shows the characteristics of patients with CRC according to the type of surgery. The average age was highest in the laparotomy group (*p* < .001), and males were dominant among all groups.

**Table 1:** Characteristics of patients with colorectal cancer based on type of surgery (n = 3,744), n (%).

	Laparotomy(n = 822)	Laparoscopy (n = 705)	Endoscopy (n = 2,217)	*p* value
Age at earliest colonoscopy (mean (SD))	74.8(10.0)	73.6(9.4)	72.7(7.7)	< .001
Sex, Male n (%)	454 (55.2)	396 (56.2)	1,456 (65.7)	< .001
Lesion (ICD-10)				< .001
Cecum (C180) and appendix (C181)	59 (7.2)	41 (5.8)	61 (1.6)	
Colon (C182–189)	562 (68.4)	529 (75.0)	1,576 (71.1)	
Rectum (C19, C20)	201 (24.5)	135 (19.2)	577 (26.0)	
In situ (D010-012)	0	0	3 (0.1)	
Preoperative long-term care needs	138 (16.8)	62 (8.8)	105 (0.5)	0.021
Requiring help 1 and 2	33 (4.0)	20 (0.3)	40 (1.8)	
Long-term care levels 1 and 2	71 (8.6)	30 (4.3)	48 (2.2)	
Level 3	18 (2.2)	6 (0.1)	9 (0.0)	
Level 4	12 (1.5)	4 (0.1)	7 (0.0)	
Level 5	2 (0.0)	2 (0.0)	1 (0.0)	
Year of diagnosis				< .001
2013	194 (23.6)	105 (14.8)	400 (18.0)	
2014	183 (22/3)	113 (16.0)	460 (20.7)	
2015	140 (17.0)	140 (19.9)	425 (19.2)	
2016	126 (15.3)	123 (17.4)	385 (17.4)	
2017	112 (13.6)	134 (19.0)	318 (14.3)	
2018 until September	67 (7.6)	90 (12.8)	229 (10.3)	
Exacerbated Long-term care needs	62 (7.5)	21 (3.0)	41 (1.8)	< .001
All course mortality ≥75 years	174 (21.2)	65 (9.2)	100 (4.5)	0.013
≤74 years	82 (10.0)	48 (6.8)	58 (2.6)	< .001

The colon was the most common cancer site, and the rectum was the second most common site among all groups. The number of beneficiaries of long-term care insurance before surgery was 138 (16.8%) in the laparotomy group, 62 in the laparoscopy group (8.8%), and 105 (0.5%) in the endoscopic surgery group (*p* = 0.021). Laparotomy was performed less frequently with time, while laparoscopy was performed more frequently after 2017. Additionally, exacerbated long-term care needs (*p* < .001) and all-cause mortality *(p* = 0.013 for those aged ≥75 years and *p* < 0.001 for those aged <75 years) were the highest in the group undergoing laparotomy, although the influence of age and cancer stage may need to be considered in interpreting this information.

### Preoperative health checkup data by surgical procedure

**Table 2** shows the results for 1,625 individuals who underwent a health checkup before their diagnosis of primary CRC. The average age and presence of hypertension, diabetes, and cardiovascular disease were higher in the laparotomy group. Other factors, including body mass index (BMI) and clinical data, showed no intergroup differences.

**Table 2:** Preoperative health checkup data by surgical procedures (n = 1,625) n (%).

	Laparotomy (n = 227)	Laparoscopy(n = 265)	Endoscopy(n = 1,132)	*p* value
**Age at earliest check-up (mean(SD))**	74.2(8.6)	70.8(8.4)	69.8(7.9)	< .001
**Sex, male**	121 (53.0)	139 (52.5)	709 (60.6)	0.001
**Smoking, yes**	34 (15.0)	29 (10.9)	141 (12.4)	0.395
**Alcohol consumption ≥40 g, yes**	17 (7.5)	21 (7.9)	111 (9.8)	0.016
**Present illness**				
**Hypertension**	119 (52.4)	112 (42.3)	543 (48.0)	0.074
**Diabetes**	28 (12.3)	21 (7.9)	119 (10.5)	0.262
**Cerebrovascular disease**	12 (6.2)	19 (8.0)	47 (5.0)	0.181
**Cardiovascular disease**	24 (12.4)	15 (6.3)	61 (6.4)	0.012
**Physical activity, yes**	75 (33.0)	91 (34.3)	385 (34.0)	0.907
BMI* (kg/m^2^) (mean(SD))	22.4 (3.3)	22.7(3.1)	23.3(3.3)	0.001
Abdominal circumference* (cm)	83.3(10.2)	83.6(9 .4)	84.7(9.3)	0.083
Systolic blood pressure* (mmHg)	135 (16)	134(19)	133(16)	0.150
Diastolic blood pressure* (mmHg)	75 (12)	76 (11)	76 (11)	0.155
Hemoglobin* (g/dL)	13.1(1.9)	13.3 (1.9)	13.9(1.5)	< .001
eGFR* (mL/min)	68.2(15.8)	69.2(13.4)	69.6(15.6)	0.539
HbA1c* NGSP 1 (%)	5.9(0.9)	5.8(0.8)	5.8(0.7)	0.484
**Exacerbated Long-term care needs**	16(7.0)	10 (3.8)	19 (1.7)	< .001
**All course mortality**	25(11.0)	63 (23.8)	94 (8.3)	< .001

### Exacerbation of long-term care needs and mortality risk

**Table 3** presents the logistic regression results for the exacerbation of long-term care needs in the laparoscopy and endoscopic surgery groups (Model 1) compared to those in the laparotomy group. The laparoscopy group showed significantly lower exacerbation of long-term care needs (OR 0.376; 0.227–0.624), while the endoscopic surgery group showed an OR of 0.231 (0.154, 0.346). Similar results were obtained for all-cause mortality. These relationships did not significantly change on adjustment for age and sex (Model 2).

**Table 3:** Odds ratio (95% confidence interval) for the exacerbation of long-term care needs and mortality for non-laparotomy treatments for initial colorectal cancer.

	Model 1 (n = 3,744)	Model 2 (n = 3,744)	Model 3 (n = 1,625)
**Exacerbation of long-term care needs**			
**Laparotomy**	(reference)	(reference)	(reference)
**Laparoscopy**	0.376 (0.227, 0.624)*	0.450 (0.266, 0.760)*	0.478 (0.393, 0.630)*
**Endoscopic surgery**	0.231 (0.154, 0.346)*	0.398 (0.259, 0.612)*	0.464 (0.367, 0.590)*
**All course mortality**			
**Laparotomy**	(reference)	(reference)	(reference)
**Laparoscopy**	0.422 (0.329, 0.542)*	0.436 (0.338, 0.563)*	0.478 (0.309, 0.740)*
**Endoscopic surgery**	0.170 (0.136, 0.211)*	0.182 (0.146, 0.228)*	0.247 (0.165, 0.370)*

Model 1: crude (Prob>chi2 = 0.00, Pseudo R2 = 0.013 for exacerbation of long term, Prob>chi2 = 0.00, Pseudo R2 = 0.033 for All course mortality)

Model 2: adjusted for age and sex (Prob>chi2 = 0.00, Pseudo R2 = 0.175 for exacerbation of long term, Prob>chi2 = 0.00, Pseudo R2 = 0.092 for All course mortality)

Model 3: adjusted for age, sex, smoking status, alcohol consumption, BMI, hemoglobin levels, cardiovascular diseases, and physical activity (Prob>chi2 = 0.00, Pseudo R2 = 0.242 for exacerbation of long term, Prob>chi2 = 0.00, Pseudo R2 = 0.124 for All course mortality)

*significant

In Model 3, the OR for long-term care was significantly lower in laparoscopic surgery than in laparotomy, with better goodness of fit of the overall model. However, the differences in beneficiaries of long-term insurance vary among groups and might affect the exacerbations of long-term needs. The risk was also significantly lower than the adjusted OR for all-cause mortality following laparoscopic surgery.

## Discussion

The laparoscopy and endoscopic surgery groups showed a significantly better long-term prognosis than the laparotomy group regarding the exacerbation of long-term care needs and all-cause mortality risk. These findings were consistent even after adjusting for sex, age, and other relevant factors. However, the endoscopic surgery group results need to be carefully interpreted because most of these patients were identified with Stage 0 and 1 CRC after screening or follow-up for polyps by colonoscopy. Moreover, the treatment strategy for CRC is determined comprehensively based on factors such as prior abdominal surgeries, the stage of progression, obesity, and overall systemic conditions, including complications. However, the KDB database does not contain information; therefore, these contraindications were not considered in this analysis.

The main strengths of this study were the large sample size collated from medical claims and long-term care data. It is generally difficult to configure natural prognoses in clinical trials. To the best of our knowledge, this is the first study to analyze post-discharge, long-term care needs, and all-cause mortality regarding CRC. Meanwhile, the total insurance benefits in 2019 (including long-term care preventive services) amounted to 837.8 billion yen in Japan [22] (equivalent to approximately 9 billion US dollars). It is important to consider quality of life (QOL), such as exacerbation of long-term care needs as well as increasing medical expenses, when patients and healthcare providers select treatments in an aging society.

Although a recent meta-analysis indicated that laparoscopy is not inferior to laparotomy [23], considering the cost-effectiveness [24], variables such as higher BMI [25], patients’ general condition, comorbidity, and age should be considered when surgical treatment is selected [26]. According to 2019 clinical practice guidelines for CRC [27], both laparotomy and laparoscopic surgery are appropriate for Stage I (T1b, T2), II, and III disease and can be adapted based on experience, practical clinician training, and patient-specific factors such as cancer site or stage. Although recommendations specific to the colon site (e.g., for ascending CRC) were not considered in this study, different indications for laparoscopic CRC should be investigated based on outcomes and related factors such as age group [28] before abdominal surgery [29]. Additionally, hospital-related factors such as the outcome measures used might affect the prognosis.

Patients reported that QOL (such as QOL reported through the 36-Item Short-Form Survey [SF36] [30], Activities of Daily Living [ADL] [31], Instrumental Activities of Daily Living [IADL] [32], or EuroQol-5D [EQ-5D] [33] instruments) was an important long-term outcome (particularly in older adults). However, to our knowledge, there are few studies regarding postoperative QOL [34, 35]. Stage IV patients who undergo colorectal surgery using more invasive procedures, such as with combined resection of other organs or those who were not indicated for surgery, may have a decreased QOL. Besides anonymized medical claims data, additional studies evaluating both clinical information (such as TNM classifications and the presence of colostomy) and long-term needs profiles (e.g., ADL and IADL scores, a comprehensive geriatric assessment [CGA] such as the Clinical Frailty Scale [36], and patient-reported QOL) are necessary to comprehensively assess the natural prognosis after CRC surgery.

This study has some limitations, including the possibility of the misclassification of primary CRC. For example, we extracted careful definitions from vague disease names in the health insurance claims data. Second, selection bias cannot be ignored because the treatment approach was driven by significant differences in patients’ conditions, cancer stage, and tumor aggressiveness. TNM classification is currently considered the most prognostic factor [37]. Clinical information regarding tumor characteristics was not available for analysis in this study, although post-surgical complications, including perforation, stoma-related obstruction [38], incisional hernia [39], or enhanced recovery care [40], were related to emergency indications and long-term prognosis. Further epidemiological research should qualify comprehensive prognosis after CRC surgery, including robot-assisted laparoscopy, which was not included in Japanese insurance [3, 4].

## Conclusion

The national health insurance claim database rarely included information on cancer stage and comorbidities, which is essential in the analysis of long-term prognosis for patients with CRC. Laparoscopy showed superior outcomes regarding long-term care needs and mortality compared to laparotomy. Our results are generalizable and should be considered in making clinical decisions.

## Supporting information

S1 Table Summary procedure code (K codes) for colorectal cancer treatment. (DOCX)

S2 Table The RECORD statement—Checklist of items extended from the STROBE statement that should be reported in observational studies using routinely collected health data. (DOCX)

## References

[1] Cancer Information Service, National Cancer Center. Vital statistics of Japan. Cancer Statistics. Tokyo, Japan: Japan Ministry of Health, Labor, and Welfare. Available from: https://ganjoho.jp/public/qa_links/report/statistics/2021_en.html.

[2] National Cancer Center, Center for Cancer Control and Information Services. Latest cancer statistics. Tokyo, Japan: Japan Ministry of Health, Labor, and Welfare. Available from: https://ganjoho.jp/public/qa_links/report/statistics/pdf/cancer_statistics_2021.pdf

[3] Ministry of Health, Labor and Welfare. The long-term care insurance system. Tokyo, Japan: Japan Ministry of Health, Labor, and Welfare. Available from: https://www.mhlw.go.jp/english/wp/wp-hw13/dl/10e.pdf.

[4] Spillman BC, Lubitz J. The effect of longevity on spending for acute and long-term care. N Engl J Med. 2000;342: 1409–1415. doi: 10.1056/NEJM200005113421906 10805827

[5] Tomita N, Yoshimura K, Ikegami N. Impact of home and community-based services on hospitalisation and institutionalisation among individuals eligible for long-term care insurance in Japan. BMC Health Serv Res. 2010;10: 345. doi: 10.1186/1472-6963-10-345 21176165 PMC3024297

[6] Utsumi T, Horimatsu T, Nishikawa Y, Hoshino N, Takahashi Y, Goto R, et al. Medical costs according to the stages of colorectal cancer: an analysis of health insurance claims in Hachioji, Japan. J Gastroenterol. 2021;56: 903–913. doi: 10.1007/s00535-021-01798-9 34215929

[7] Kurita A, Nakamura Y. Health check-up results, death, and occurrence of the need for nursing care among Japanese older adults: analysis using the Kokuho Database system. Nihon Koshu Eisei Zasshi. 2023;70: 16–26. doi: 10.11236/jph.22-037 36058876

[8] Ministry of Health, Labour and Welfare. Annual Report of in-Hospital Cancer Registration. 2020 version. Tokyo, Japan: Japan Ministry of Health, Labor, and Welfare. Available from: https://ganjoho.jp/public/qa_links/report/hosp_c/pdf/2020_report.pdf.

[9] Clinical Outcomes of Surgical Therapy Study Group, Nelson H, Sargent DJ, Wieand HS, Fleshman J, Anvari M, et al. A comparison of laparoscopically assisted and open colectomy for colon cancer. N Engl J Med. 2004;350: 2050–2059. doi: 10.1056/NEJMoa032651 15141043

[10] Fleshman J, Sargent DJ, Green E, Anvari M, Stryker SJ, Beart R, et al. Laparoscopic colectomy for cancer is not inferior to open surgery based on 5-year data from the COST Study Group trial. Ann Surg. 2007;246: 655–664. doi: 10.1097/SLA.0b013e318155a762 17893502

[11] Kitano S, Inomata M, Mizusawa J, Katayama H, Watanabe M, Yamamoto S, et al. Survival outcomes following laparoscopic versus open D3 dissection for stage II or III colon cancer (JCOG0404): a phase 3, randomised controlled trial. Lancet Gastroenterol Hepatol. 2017;2(4): 261–268. doi: 10.1016/S2468-1253(16)30207-2 28404155

[12] Tamakoshi A, Nakamura K, Ukawa S, Okada E, Hirata M, Nagai A, et al. Characteristics and prognosis of Japanese colorectal cancer patients: the BioBank Japan Project. J Epidemiol. 2017;27: S36–S42. doi: 10.1016/j.je.2016.12.004 28214186 PMC5350596

[13] Ikegami N, Yoo BK, Hashimoto H, Matsumoto M, Ogata H, Babazono A, et al. Japanese universal health coverage: evolution, achievements, and challenges. Lancet. 2011;378: 1106–1115. doi: 10.1016/S0140-6736(11)60828-3 21885107

[14] Higashizono K, Nakatani E, Hawke P, Fujimoto S, Oba N. Risk factors for gallstone disease onset in Japan: Findings from the Shizuoka Study, a population-based cohort study. PLoS One. 2022;17: e0274659. doi: 10.1371/journal.pone.0274659 36584097 PMC9803237

[15] Tamaru Y, Oka S, Tanaka S, Nagata S, Hiraga Y, Kuwai T, et al. Long-term outcomes after treatment for T1 colorectal carcinoma: a multicenter retrospective cohort study of Hiroshima GI Endoscopy Research Group. J Gastroenterol. 2017;52: 1169–1179. doi: 10.1007/s00535-017-1318-1 28194526

[16] Goto H, Nakatani E, Yagi H, Moriki M, Sano Y, Miyachi Y. Late-onset development of psoriasis in Japan: a population-based cohort study. JAAD Int. 2021;2: 51–61. doi: 10.1016/j.jdin.2020.10.011 34409354 PMC8362311

[17] Nomura S, Sakamoto H, Rauniyar SK, Shimada K, Yamamoto T, Kohsaka S, et al. Analysis of the relationship between the HbA1c screening results and the development and worsening of diabetes among adults aged over 40 Years: a 4-year follow-up study of 140,000 people in Japan–the Shizuoka study. BMC Public Health. 2021;21: 1880. doi: 10.1186/s12889-021-11933-z 34663286 PMC8524880

[18] Nishimura S, Kumamaru H, Shoji S, Nakatani E, Yamamoto H, Ichihara N, et al. Assessment of coding-based frailty algorithms for long-term outcome prediction among older people in community settings: a cohort study from the Shizuoka Kokuho Database. Age Ageing. 2022;51: afac009. doi: 10.1093/ageing/afac009 35231096 PMC9077119

[19] Iwagami M, Tamiya N. The long-term care insurance system in Japan: past, present, and future. JMA J. 2019;2: 67–69. doi: 10.31662/jmaj.2018-0015 33681515 PMC7930803

[20] Nakatani E, Tabara Y, Sato Y, Tsuchiya A, Miyachi Y. Data resource profile of Shizuoka Kokuho Database (SKDB) using integrated health- and care-insurance claims and health checkups: the Shizuoka Study. J Epidemiol. 2022;32: 391–400. doi: 10.2188/jea.JE20200480 33518592 PMC9263618

[21] Benchimol EI, Smeeth L, Guttmann A, Harron K, Moher D, Petersen I, et al. The REporting of studies Conducted using Observational Routinely collected health Data (RECORD) statement. PLOS Med. 2015;12: e1001885. doi: 10.1371/journal.pmed.1001885 26440803 PMC4595218

[22] Xu K, Saksena P, Holly A. The Determinants of Health Expenditure: a Country-Level Panel Data Analysis Cited July 2022. Geneva, Switzerland: World Health Organization (World Health Organization); 2011;26:1–28

[23] Ishiyama Y, Tachimori Y, Harada T, Mochizuki I, Tomizawa Y, Ito S, et al. Oncologic outcomes after laparoscopic versus open multivisceral resection for local advanced colorectal cancer: A meta-analysis. Asian J Surg. 2023;46: 6–12. doi: 10.1016/j.asjsur.2022.02.047 35568616

[24] Hirpara DH O’Rourke C, Azin A, Quereshy FA, Wexner SD, Chadi SA. Impact of BMI on adverse events after laparoscopic and open surgery for rectal cancer. J Gastrointest Cancer. 2022;53: 370–379. doi: 10.1007/s12029-021-00612-2 33660225

[25] Guillou PJ, Quirke P, Thorpe H, Walker J, Jayne DG, Smith AM, et al. Short-term endpoints of conventional versus laparoscopic-assisted surgery in patients with colorectal cancer (MRC CLASICC trial): multicentre, randomised controlled trial. Lancet. 2005;365: 1718–1726. doi: 10.1016/S0140-6736(05)66545-2 15894098

[26] Hashiguchi Y, Muro K, Saito Y, Ito Y, Ajioka Y, Hamaguchi T, et al. Japanese Society for Cancer of the Colon and Rectum (JSCCR) guidelines 2019 for the treatment of colorectal cancer. Int J Clin Oncol. 2020;: 1–42. doi: 10.1007/s10147-019-01485-z 31203527 PMC6946738

[27] Chern YJ, Hung HY, You JF, Hsu YJ, Chiang JM, Hsieh P, et al. Advantage of laparoscopy surgery for elderly colorectal cancer patients without compromising oncologic outcome. BMC Surg. 2020;20: 294. doi: 10.1186/s12893-020-00967-6 33228630 PMC7686695

[28] Sueda T, Tei M, Nishida K, Yoshikwa Y, Matsumura T, Koga C, et al. Impact of prior abdominal surgery on short-term outcomes following laparoscopic colorectal cancer surgery: a propensity score-matched analysis. Surg Endosc. 2022;36: 4429–4441. doi: 10.1007/s00464-021-08794-3 34716479

[29] Kim CW, Park YY, Hur H, Min BS, Lee KY, Kim NK. Cost analysis of single-incision versus conventional laparoscopic surgery for colon cancer: A propensity score-matching analysis. Asian J Surg. 2020;43: 557–563. doi: 10.1016/j.asjsur.2019.06.012 31345655

[30] Ware JE Jr, Kosinski M, Gandek B, Aaronson NK, Apolone G, Bech P, et al. The factor structure of the SF-36 Health Survey in 10 countries: results from the IQOLA Project. International quality of life assessment. J Clin Epidemiol. 1998;51: 1159–1165. doi: 10.1016/s0895-4356(98)00107-3 9817133

[31] Katz S, Ford AB, Moskowitz RW, Jackson BA, Jaffe MW. Studies of illness in the aged: the Index of ADL: a standardized measure of biological and psychosocial function. JAMA. 1963;185: 914–919. doi: 10.1001/jama.1963.03060120024016 14044222

[32] Bottari CL, Dassa C, Rainville CM, Dutil E. The IADL Profile: development, content validity, intra- and interrater agreement. Can J Occup Ther. 2010;77: 90–100. doi: 10.2182/cjot.2010.77.2.5 20464894

[33] Brooks R. EuroQol: the current state of play. Health Policy. 1996;37: 53–72. doi: 10.1016/0168-8510(96)00822-6 10158943

[34] Sakamoto K, Tsukamoto R, Kawano S, Kawai M, Niwa K, Ishiyama S, et al. Minimally invasive surgery for colorectal cancer. Juntendo J. 2017;63:384–392. doi: 10.14789/jmj.63.384

[35] Colon Cancer Laparoscopic or Open Resection Study Group, Buunen M, Veldkamp R, Veldkamp R, Hop WC, Kuhry E et al.Survival after laparoscopic surgery versus open surgery for colon cancer: long-term outcome of a randomised clinical trial. Lancet Oncol. 2009;10: 44–52. doi: 10.1016/S1470-2045(08)70310-3 19071061

[36] Rockwood K, Song X, MacKnight C, Bergman H, Hogan DB, McDowell I, et al. A global clinical measure of fitness and frailty in elderly people. CMAJ. 2005;173: 489–495. doi: 10.1503/cmaj.050051 16129869 PMC1188185

[37] Fortea-Sanchis C, Martínez-Ramos D, Escrig-Sos J. The lymph node status as a prognostic factor in colon cancer: comparative population study of classifications using the logarithm of the ratio between metastatic and nonmetastatic nodes (LODDS) versus the pN-TNM classification and ganglion ratio systems. BMC Cancer. 2018;18: 1208. doi: 10.1186/s12885-018-5048-4 30514228 PMC6280498

[38] Maemoto R, Tsujinaka S, Miyakura Y, Fukuda R, Kakaizawa N, Takeami T, et al. Risk factors and management of stoma-related obstruction after laparoscopic colorectal surgery with diverting ileostomy. Asian J Surg. 2021;44: 1037–1042. doi: 10.1016/j.asjsur.2021.01.002 Epub February 3 2021. . 33549406

[39] Khor SN, Cheok SHX, Sultana R, Tan EKW. Incidence of incisional hernia after major colorectal cancer surgery and analysis of associated risk factors in Asian population: is laparoscopy any better? Asian J Surg. 2023;46: 99–104. doi: 10.1016/j.asjsur.2022.01.029 Epub February 11 2022. . 35165026

[40] Seow-En I, Wu J, Yang LWY, Tan JSQ, Seah AWH, Foo FJ, et al. Results of a colorectal enhanced recovery after surgery (ERAS) programme and a qualitative analysis of healthcare workers’ perspectives. Asian J Surg. 2021;44: 307–312. doi: 10.1016/j.asjsur.2020.07.020 Epub August 27 2020. . 32863145

