# Supplementary material for: Longitudinal analysis of long-term outcomes of colorectal cancer after laparotomy and laparoscopic surgery: The Shizuoka study
Source: PLoS One. 2023 Nov 17;18(11):e0294589. doi: 10.1371/journal.pone.0294589 (PMC10656028; doi:10.1371/journal.pone.0294589)
Supplement: S1 Table — (DOCX) [file pone.0294589.s001.docx]

S1 Table Summary procedure code (K codes) for colorectal cancer treatment

| **procedure code** | **Name of medical procedure** |
| --- | --- |
| **Laparotomy** | |
| K719-0 | Colectomy |
| K720-0 | Colon tumor, colon diverticulectomy, colon polypectomy  Colon tumor removal (including ileocecal tumor removal) |
| K740-0 | Rectal resection/amputation |
| **Laparoscopic surgery** | |
| K719-2 | Laparoscopic colectomy |
| K719-3 | Laparoscopic malignant colectomy |
| K740-2 | Laparoscopic rectal resection/amputation |
| **Endoscopic surgery** | |
| K721 | Endoscopic colon polyp, mucosal resection |
| K721-2 | Endoscopic colorectal polypectomy |
| K721-4 | Early-stage malignant tumor colorectal submucosal dissection |
